# Supplementary material for: Single-cell reconstruction reveals input patterns and pathways into corticotropin-releasing factor neurons in the central amygdala in mice
Source: Commun Biol. 2022 Apr 6;5:322. doi: 10.1038/s42003-022-03260-9 (PMC8986827; doi:10.1038/s42003-022-03260-9)
Supplement: Supplementary file 2 — Supplementary Information [file 42003_2022_3260_MOESM2_ESM.pdf]

Supplementary Information

Figures

Supplementary Fig. 1: Representative images of experimental controls.

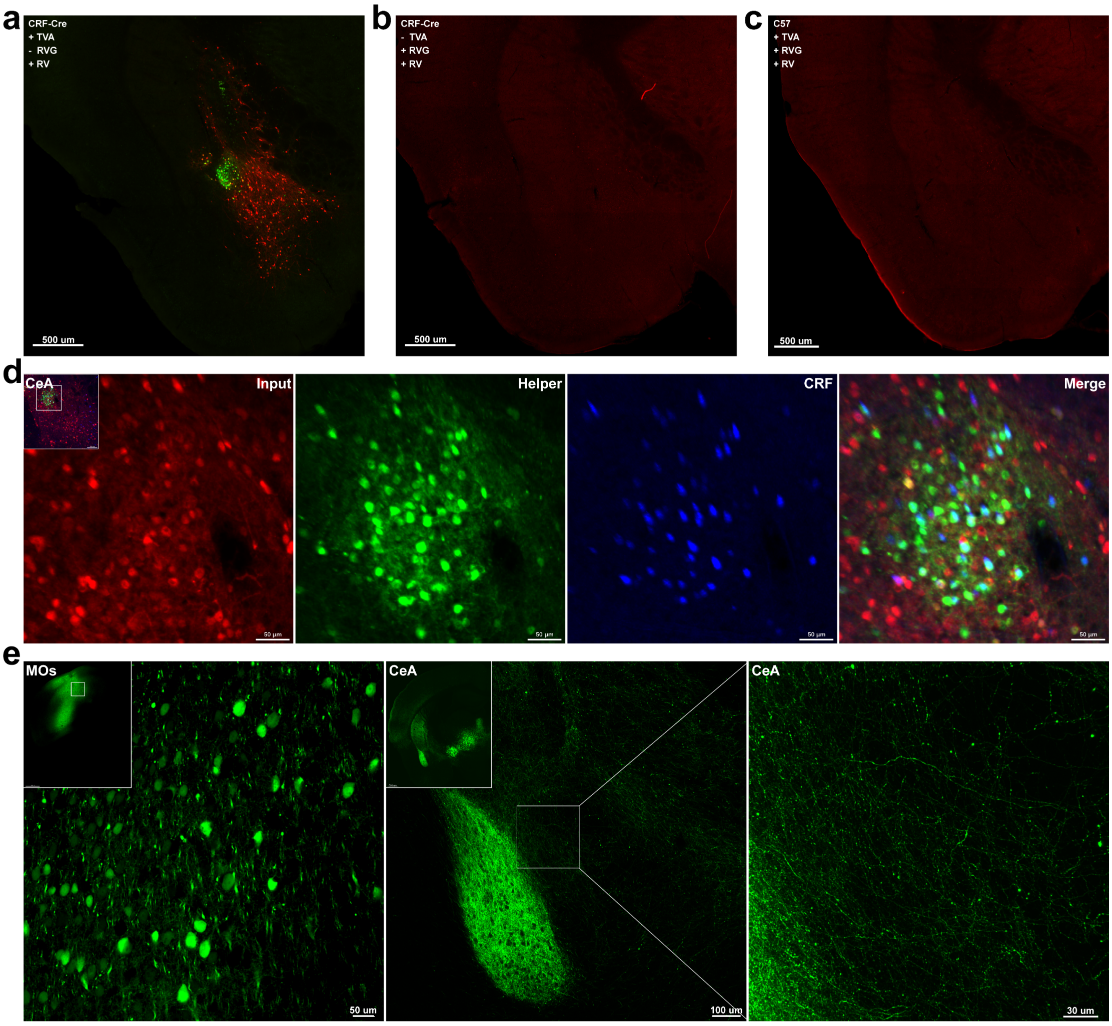

a To confirm the necessity of rabies glycoprotein G (RG) to rabies virus transsynaptic transmission, only one of the two helper viruses (AAV-DIO-TVA-EGFP, green) and helper-dependent rabies virus (red) were injected into the CeA of CRF-Cre mice, which resulted in neurons that coexpressed both EGFP and DsRed without extrinsic input only expressed as DsRed. This result indicated that RG was a key component for rabies virus to retrogradely infect from starter neurons. (scale bar: 500 μm) **b** Similarly, only injecting one of the two helper viruses (AAV-DIO-RG) and helper-dependent rabies virus did not result in any DsRed-labeled neurons, indicating that the receptor of avian sarcoma leucosis virus envelope protein (TVA) was necessary for rabies virus to infect starter neurons. (scale bar: 500 μm) **c** Injecting both of the two helper viruses (AAV-DIO-TVA-EGFP, AAV-DIO-RG) and helper-dependent rabies virus into wild-type mice also did not result in DsRed-labeled neurons, indicating that all helper viruses expressed all components strictly depending on the expression of Cre recombinase. (scale bar: 500 μm) **d** To further confirm the specificity of the neuronal types of starter neurons, we performed FISH

to identify the neuron-subtype of starter neurons. Most of the starter neurons were positive for the CRF mRNA probe, which indicated that the starter neurons were CRF-expressing neurons and that the CRF-Cre line that we used had a high specificity. Representative tile scan image in the CeA shows that most of the starter neurons that coexpressed helper viruses (green) and rabies virus (red) were CRF-positive neurons (blue). (scale bar: 150 μm and 50 μm respectively) **e** An AAV expressing EGFP driven by CaMKII were injected in secondary motor area, where the CaMKII-expressing inputs mainly distributed. (scale bar: 50 μm) The terminal fibers can be clearly observed in CeA, indicating that there were inputs expressing CaMKII projecting to central amygdala. (scale bar: 100 μm and 30 μm respectively)

Supplementary Fig. 2: Most starter neurons were located in the CeA.

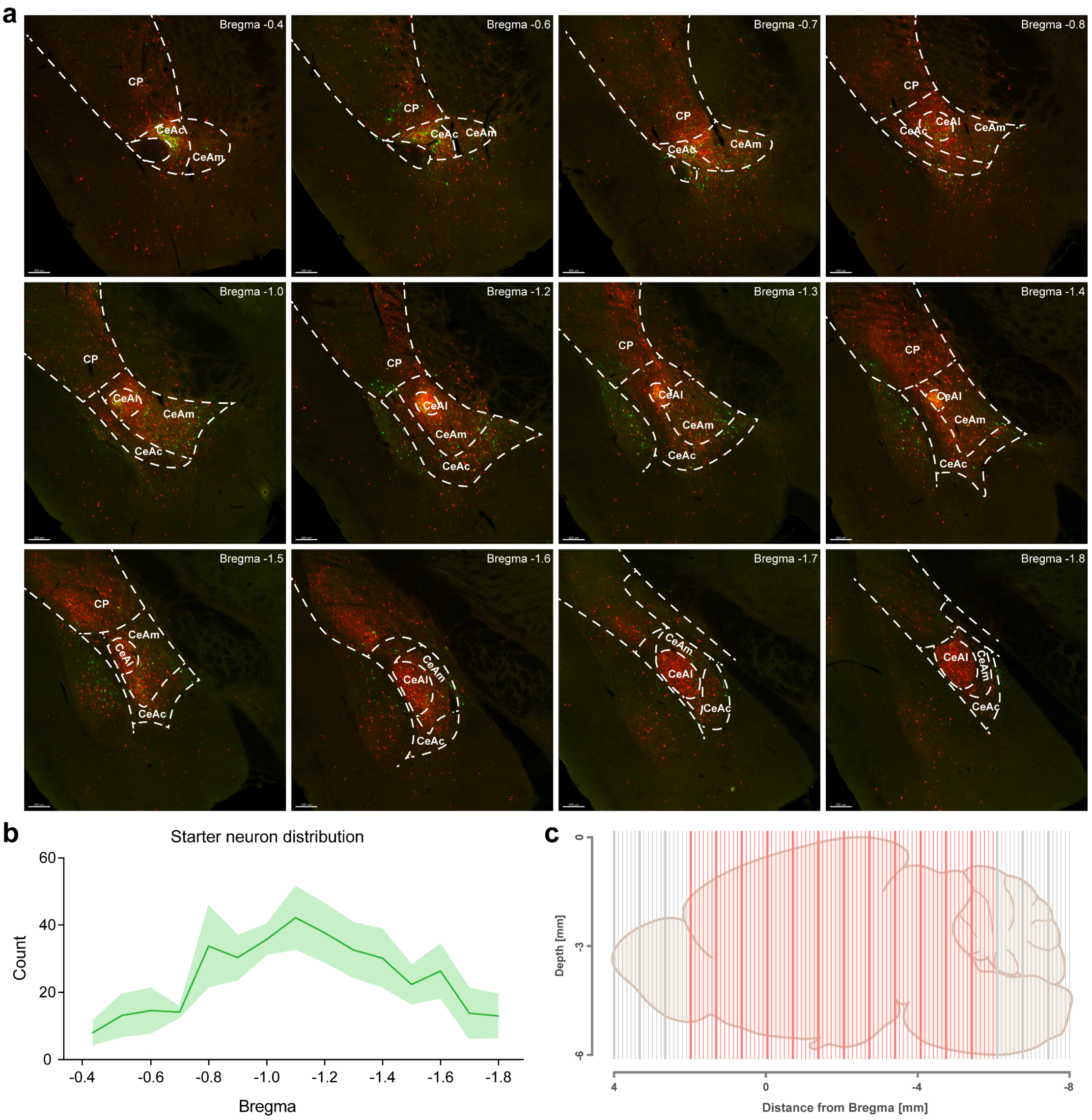

a To verify the reliability of the approach in every tracing case, the whole brain of each CRF-Cre mouse in the virus-tracing group was sectioned to examine the morphological distribution of starter neurons in the amygdala. Representative distribution of starter neurons in 50-μm serial-scanned images. The position of bregma is shown in the upper-right corner to indicate the interval between each slice. Abbreviations: CeAc: central amygdalar nucleus, capsular part, CeAl: central amygdalar nucleus, lateral part, CeAm: central amygdalar nucleus, medial part, CP, caudoputamen. (scale bar: 200 μm) **b** The distribution of starter neurons, from anterior to posterior, within the CeA. Most of the starter neurons (67.87 ± 3.31%) were in the posterior and middle parts of the CeA. The line refers to the mean number of the starters, and the shadow refers to the corresponding s.e.m. value. **c** Cartoon illustration of a brain slices (thin lines) and the representative images are shown in Fig. 2a (thick lines).

Supplementary Fig. 3: The illustrations of the distribution and morphology of inputs.

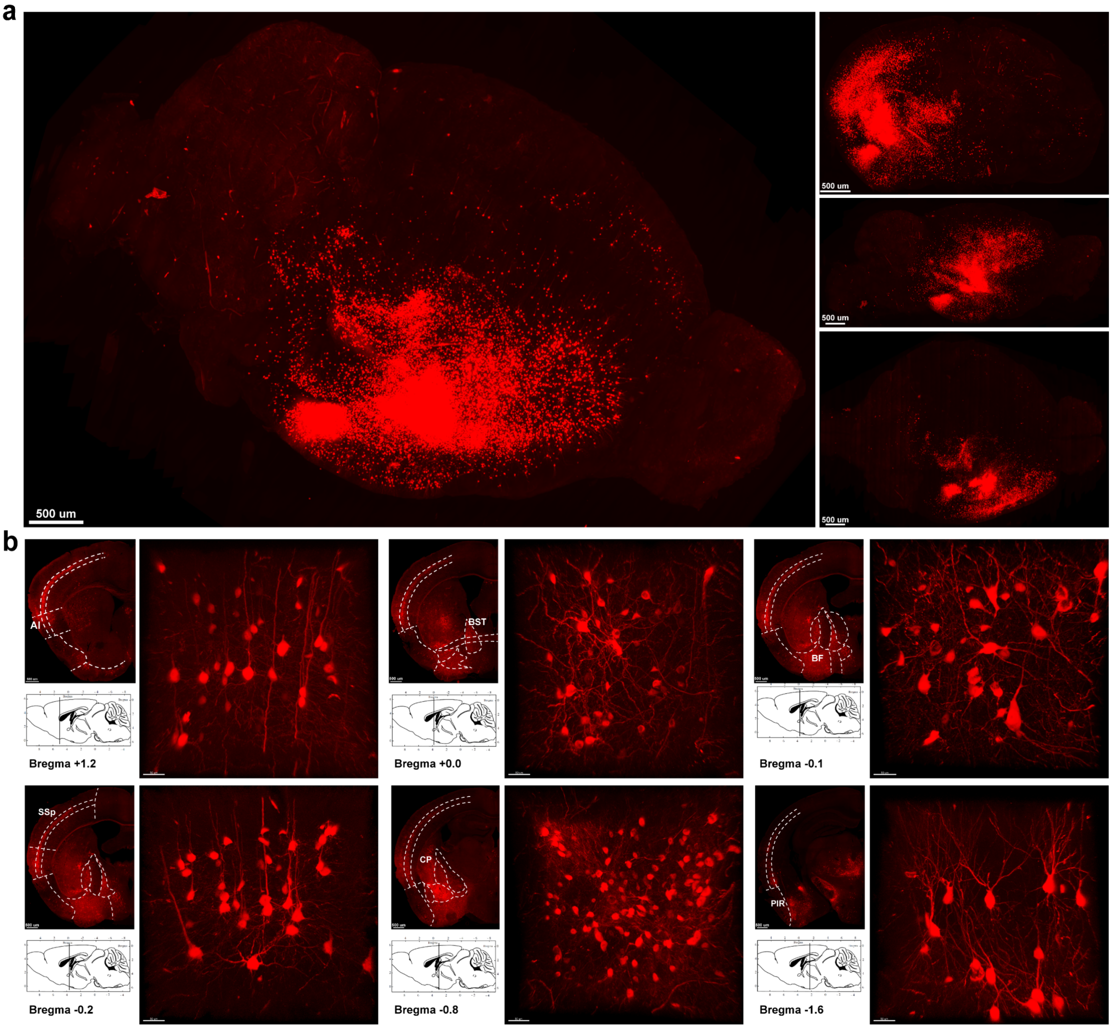

a Three-dimensional reconstructed mouse brain showing all inputs at the whole-brain scale. The front view, side view, and top view of the reconstructed brain show that the inputs were concentrated in the forebrain (scale bar: 500 μm). **b** Representative morphologies of input neurons in main input regions including the agranular insular area (AI), bed nuclei of the stria terminalis (BST), basal forebrain (BF), somatosensory area (SS), caudoputamen (CP), and piriform area (PIR) (scale bar in tile-scan images: 500 μm; scale bar in detailed images: 50 μm).

Supplementary Fig. 4: Distributions of CaMKII and GAD1-expressing inputs.

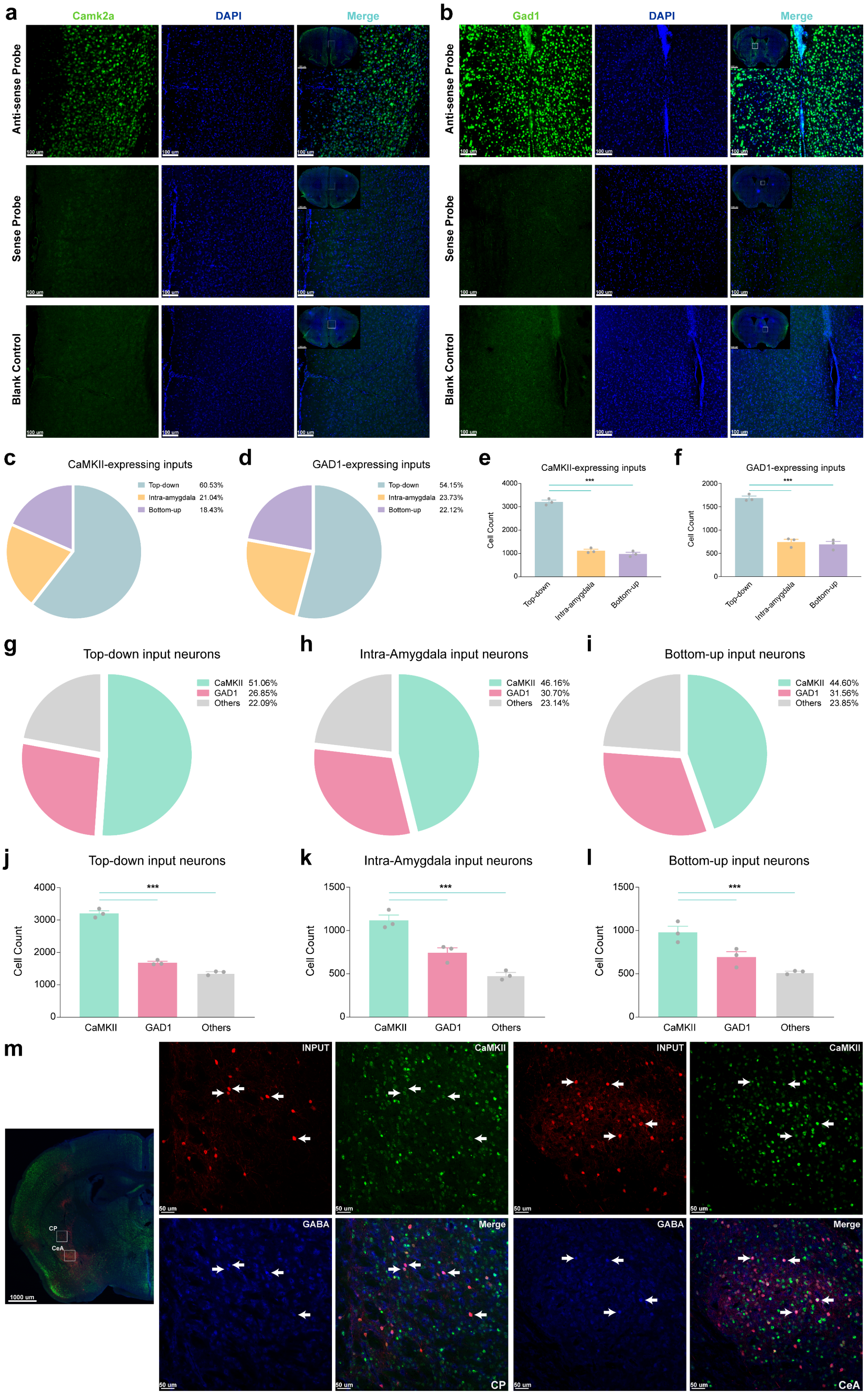

a Validation of CaMKII probes. Anti-sense, sense and blank control probes of CaMKII merged with DAPI. There were positive signals in the prefrontal cortex only tested by anti-sense probes. (scale bar: 100 μm) **b** Validation of GAD1 probes. Anti-sense, sense and blank control probes of GAD1 merged with DAPI. There were positive signals in the lateral septal nucleus only tested by anti-sense probes. (scale bar: 100 μm) **c** CaMKII-expressing inputs were concentrated in the top-down group (60.53 ± 1.12%). **d** GAD1-expressing inputs were also concentrated in the top-down group (54.15 ± 2.11%). **e** The numbers of CaMKII-expressing inputs in the top-down group were significantly larger than those of the other two groups (F [2, 6] = 313.6, $p < 0.001$). **f** The numbers of GAD1-expressing inputs in the top-down group were significantly larger than those of the other two groups (F [2, 6] = 102.7, $p < 0.001$). **g** Proportions of CaMKII- (51.06 ± 1.17%) and GAD1-expressing inputs (26.85 ± 0.72%) in top-down groups. **h** Proportions of CaMKII- (46.16 ± 1.58%) and GAD1-expressing inputs (30.70 ± 1.15%) in intra-amygdala groups. **i** Proportions of CaMKII- (44.60 ± 0.53%) and GAD1-expressing inputs (31.56 ± 1.21%) in bottom-up groups. **j** The number of CaMKII-positive input neurons was significantly larger than the number of GAD1-positive input neurons and others in the top-down (F [2, 6] = 308.0, $p < 0.0001$) groups. **k** The number of CaMKII-positive inputs was significantly larger than the number of GAD1-positive inputs and others in the intra-amygdala (F [2, 6] = 37.36, $p = 0.0004$) groups. **l** The number of CaMKII-positive inputs was significantly larger than the number of GAD1-positive inputs and others in the bottom-up (F [2, 6] = 18.10, $p = 0.0029$) groups. **m** The input neurons in CP and CeA were labeled by both CaMKII FISH probe and anti-GABA antibody, and several CaMKII-expressing inputs were colocalized with the GABA immunofluorescent both in CP and CeA, indicating that the CaMKII is not an accurate marker of excitatory neurons in subcortical regions. (scale bar: 1000 μm and 50 μm respectively) Data are presented as the mean ± s.e.m., N = 3, one-way ANOVA with Tukey correction.

Supplementary Fig. 5: Gallery of 48 reconstructed inputs arranged with respect to somatic depth and the layer of cortex.

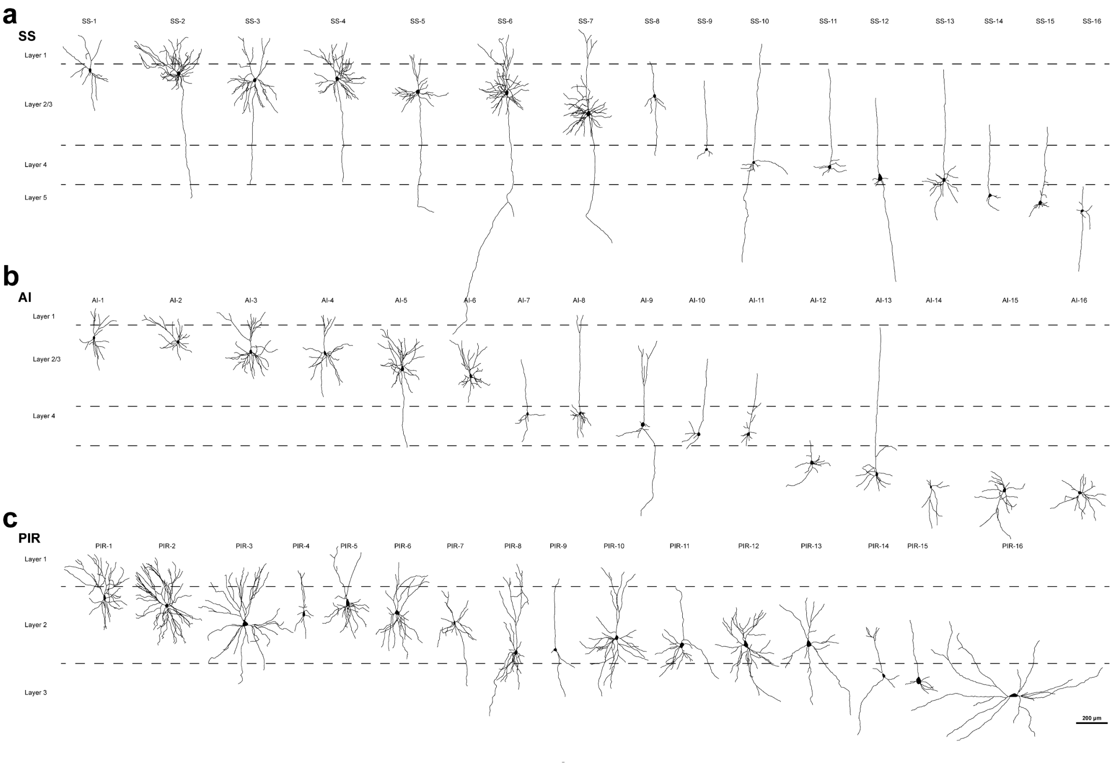

a Gallery of 16 input neurons in somatosensory area (SS). There were 8 input neurons in layer 2/3, 5 input neurons in layer 4 and 3 input neurons in layer 5. Generally, the inputs in deeper layers had simpler morphological structures. **b** Gallery of 16 input neurons in agranular insular area (AI). There were 6 input neurons in layer 2/3, 5 input neurons in layer 4 and 5 input neurons in layer 5. **c** Gallery of 16 input neurons in piriform area (PIR). There were 13 input neurons in layer 2, 3 input neurons in layer 4 and 5 input neurons in layer 5. The morphological structure of inputs in piriform area was more complex than those in somatosensory area and agranular insular area. (scale bar: 200 μm)

Supplementary Fig. 6: Eleven morphological parameters of the three input clusters in the SS, AI, and PIR.

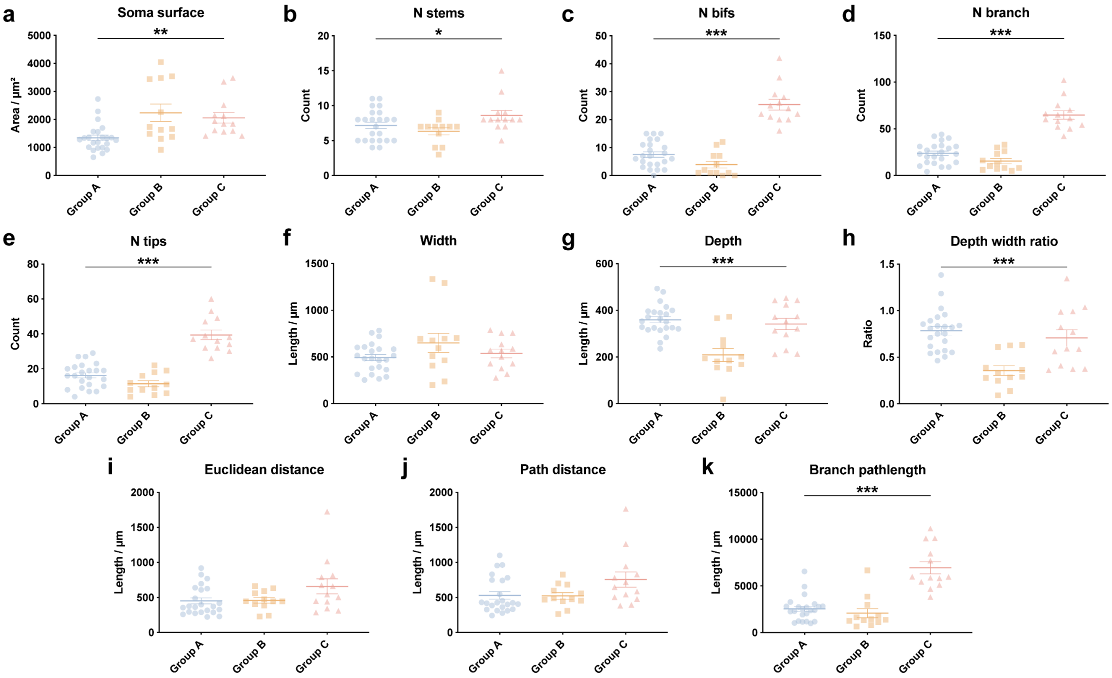

a–e Significant differences were observed in terms of soma surface (a, F [2, 45] = 7.635, p = 0.0014) and the numbers of stems (b, F [2, 45] = 3.550, p = 0.0370), bifurcations (c, F [2, 45] = 63.89, p < 0.0001), branches (d, F [2, 45] = 59.55, p < 0.0001), and tips (e, F [2, 45] = 51.45, p < 0.0001) among these groups. **f** There was no significant difference in width (F [2, 45] = 1.971, p = 0.1512) among these groups. **g–h** There were significant differences in the depth (g, F [2, 45] = 14.99, p < 0.0001) and the depth width ratio (h, F [2, 45] = 12.75, p < 0.0001) among these groups. **i–j** There were no notable differences in the Euclidean distance (i, F [2, 45] = 3.038, p = 0.0579) or path distance (j, F [2, 45] = 3.193, p = 0.0505) among these groups. **k** Significant differences were also found in the branch path length (F [2, 45] = 32.99, p < 0.0001) among these groups. All data are shown as the mean ± s.e.m., N = 48. (** p < 0.01 and *** p <0.001 via one-way ANOVA with Tukey correction).

Supplementary Fig. 7: Registration of 3D reconstructed inputs traced in the SS, MD, and PAG in a standardized mouse brain reference atlas.

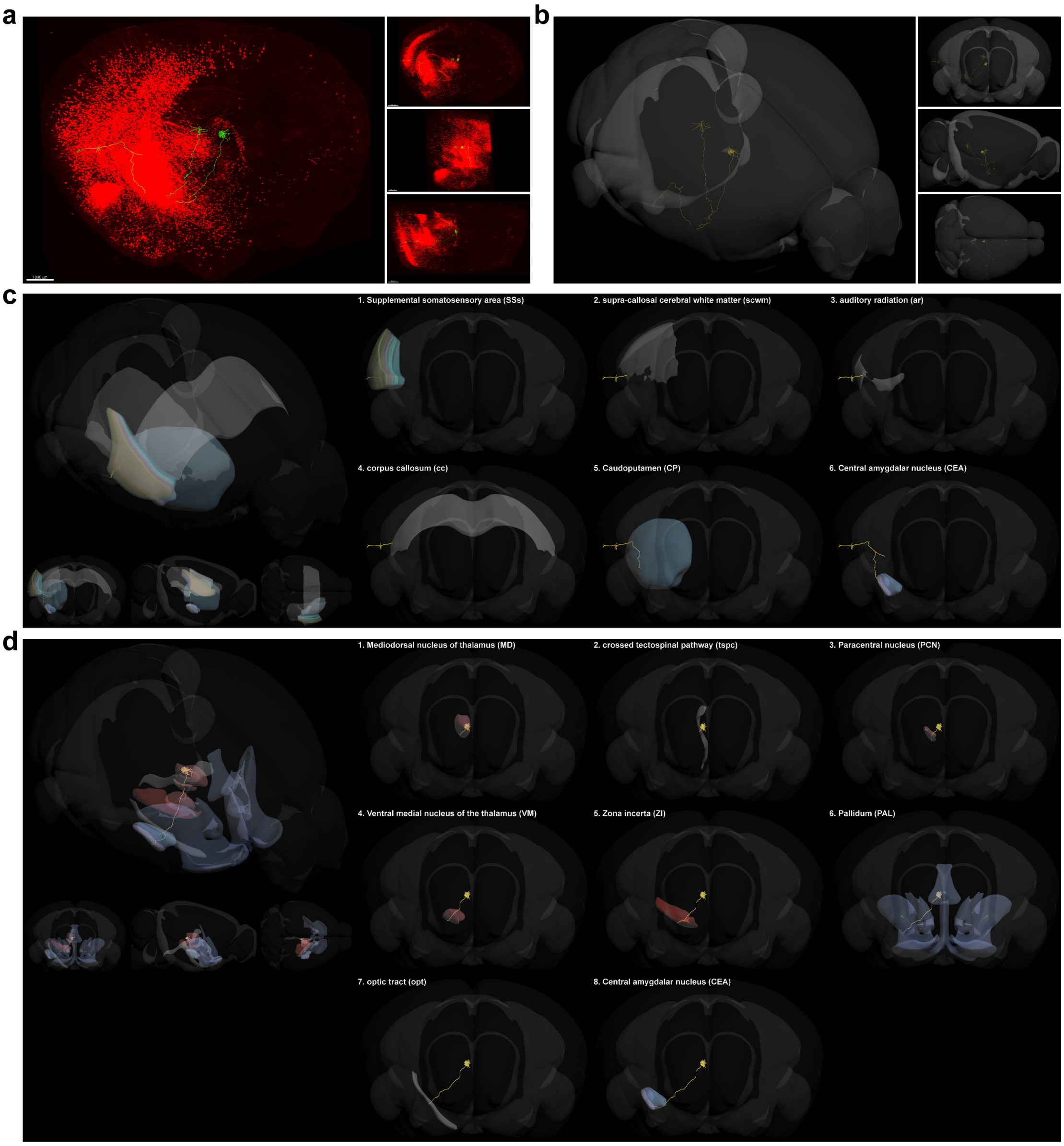

a Three input neurons (red) in the somatosensory area (SS), mediodorsal nucleus of thalamus (MD), and periaqueductal gray (PAG) were traced in 3D light-sheet-imaged brain slices. The front view, side view, and top view of the 3D image are shown. (scale bar: 1000 μm) **b** The three traced input neurons (yellow) were extracted from the 3D image and displayed in an outline of the standardized mouse brain reference atlas. The front view, side view, and top view of the outline with its neurons are shown. **c** A detailed pathway of an input neuron in the somatosensory area is shown in the 3D reference atlas with its three views. The brain regions that the input neurons passed through were the somatosensory area, supra-callosal cerebral white matter (scwm), auditory radiation (ar), corpus callosum (cc), caudoputamen (CP), and CeA sequentially, which are specifically shown in the serial images. **d** An input neuron in the mediodorsal nucleus of thalamus is shown in the 3D reference atlas with the brain regions

it passed through, which were the mediodorsal nucleus of thalamus, crossed tectospinal pathway (tspc), Paracentral nucleus (PCN), Ventral medial nucleus of the thalamus (VM), Zona incerta (ZI), Pallidum (PAL), optic tract (opt), and CeA sequentially.

Supplementary Fig. 8: Input fibers have connections in their en passant structures.

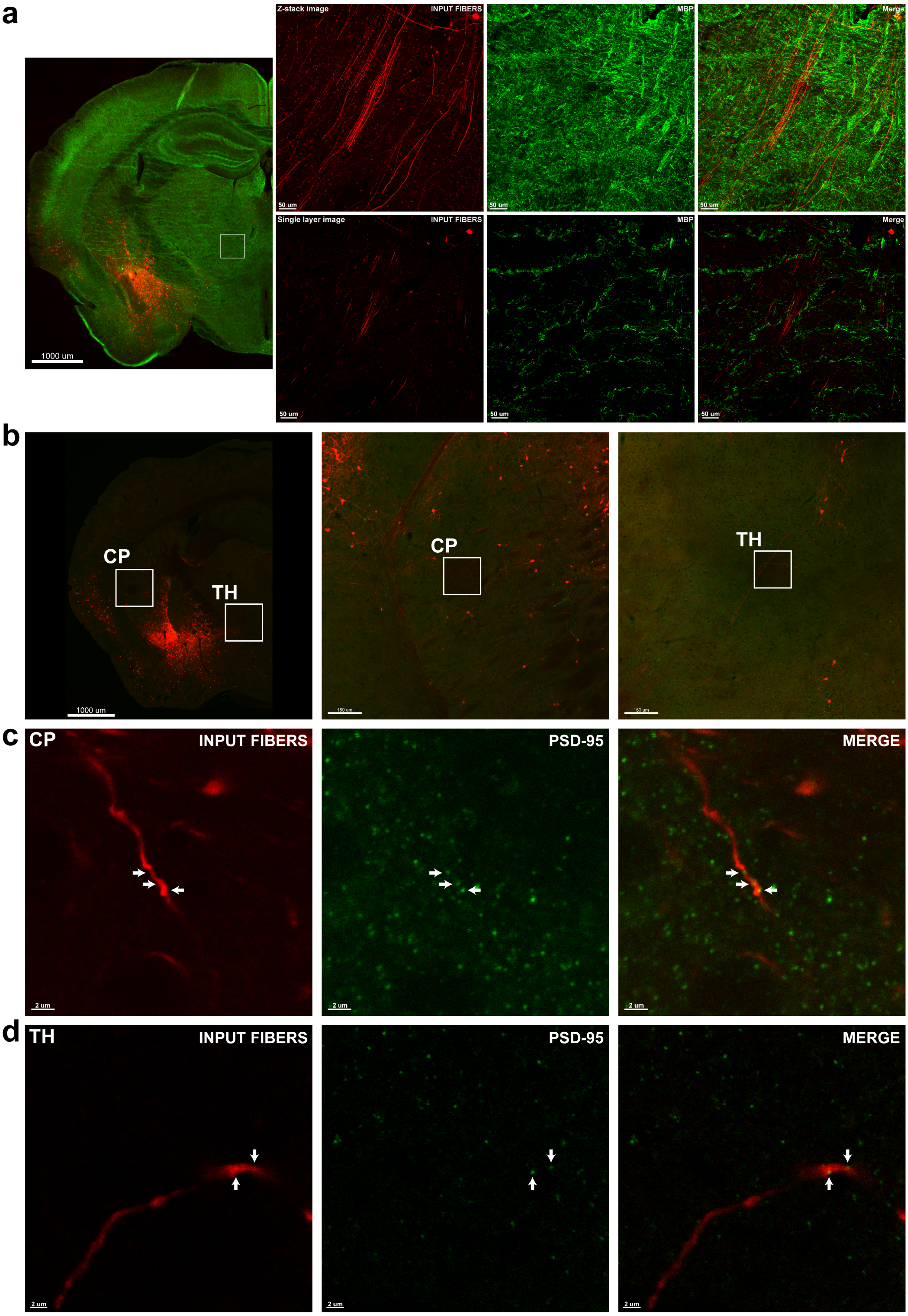

a The Z-stack image and a representative layer in this Z-stack showed that the input fibers (red) and the myelin basic protein (green) were not colocalized, indicating that the passing axon was not wrapped in myelin. (scale bar: 1000μm and 50 μm respectively) **b** A representative image showing the input fibers

(red) and the postsynaptic density protein 95 (PSD-95, green) in the highlighted structures (scale bar: 1000μm and 150 μm respectively). **c** A single-layer image demonstrating that the input fibers (red) were colocalized with PSD-95 at the CP (scale bar: 2um). **d** The input fibers (red) were also colocalized with PSD-95 at the thalamus in a single-layer image. These results indicated that the long-range projecting fibers had connections in their en passant structures, and these connections may modulate the information transmitted in the fibers (scale bar: 2um).

Supplementary Fig. 9: All the structures that the input fibers pass through in a flowchart.

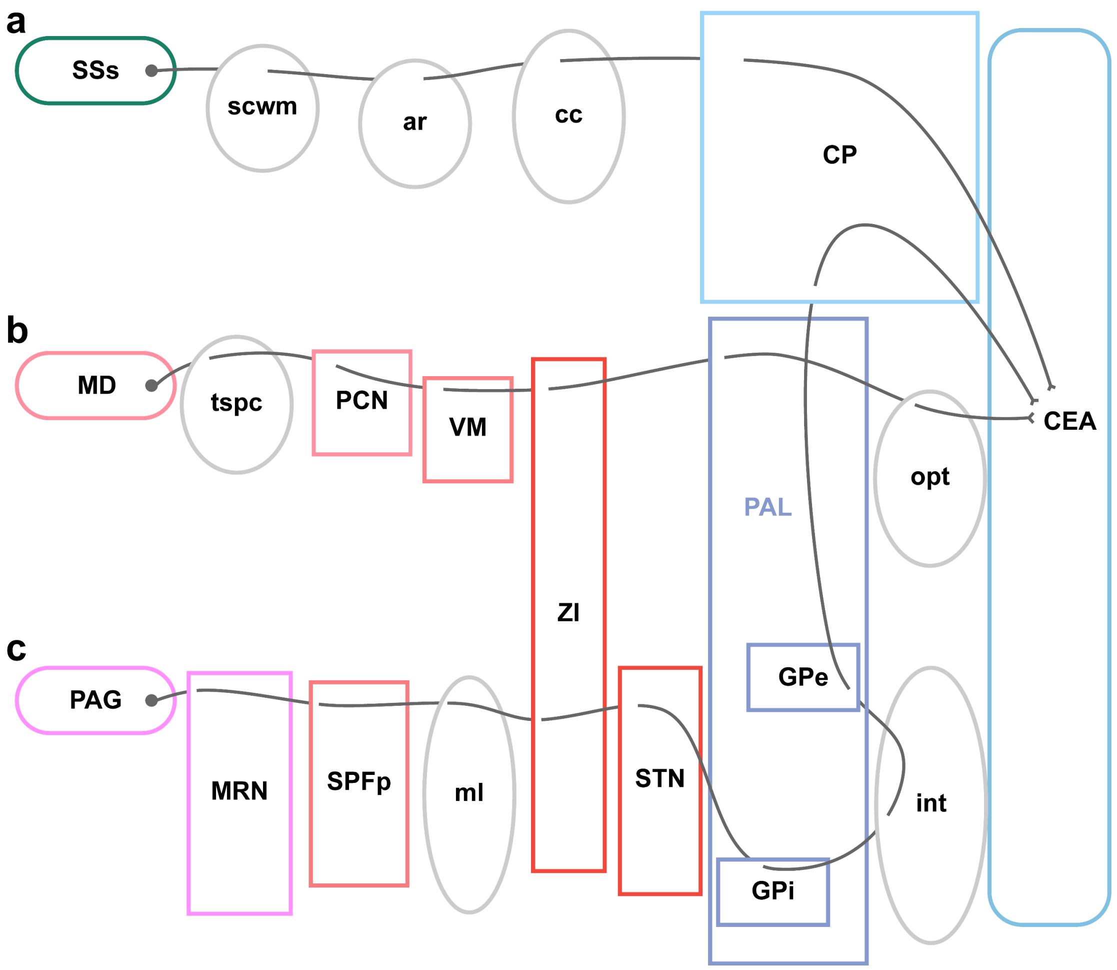

a The input neuron located in the supplemental somatosensory area (SSs) had the shortest input pathway, directly crossing three white matters and reaching the CeA only through the caudoputamen (CP). **b** The mediodorsal nucleus of thalamus (MD) transmits cognitive memory-related information, while its emitted fibers pass through the paracentral nucleus (PCN) in the thalamus, which also receives input from cortical information and corrects the afferent information from the mediodorsal nucleus of thalamus. Then, the ventral medial nucleus of the thalamus (VM), zona incerta (ZI) and pallidum (PAL) that the fiber passes through are all highly correlated with motor function. **c** The periaqueductal gray (PAG) conveys information involved in motivated behavior and processes controlling, and midbrain reticular nucleus (MRN) participates in cognitive and mood-related functions. while subparafascicular nucleus, parvicellular part (SPFp) participates in sexually related and conditioned fear behaviors, which are both highly involved in the amygdala. Then, the fiber undergoes a series of modifications from several motion-related structures before finally entering CeA.

Tables

Supplementary Table 1. Subregions and their abbreviations in the top-down groups.

Subregions	Abbreviation
Nucleus accumbens	ACB
Agranular insular area	AI
Bed nuclei of the stria terminalis	BST
Caudoputamen	CP
Endopiriform nucleus	EP
Frontal pole	FRP
Fundus of striatum	FS
Globus pallidus	GP
Gustatory areas	GU
Magnocellular nucleus	MA
Mediodorsal nucleus of thalamus	MD
Primary motor area	MO
Diagonal band nucleus	NDB
Nucleus of the lateral olfactory tract	NLOT
Orbital area	ORB
Olfactory tubercle	OT
Piriform-amygdalar area	PAA
Piriform area	PIR
Paraventricular nucleus of the thalamus	PVT
Substantia innominata	SI
Somatosensory area	SS
Visceral area	VISC
Cortical amygdalar area	COA

Supplementary Table 2. Subregions and their abbreviations in the intra-amygdala groups.

Subregions	Abbreviation
Anterior amygdalar area	AAA
Basolateral amygdalar nucleus, anterior part	BLA
Basomedial amygdalar nucleus	BMA
Central amygdalar nucleus	CEA
Medial amygdalar nucleus	MEA

Supplementary Table 3. Subregions and their abbreviations in the bottom-up groups.

Subregions	Abbreviation
Auditory areas	AUD
Dorsal nucleus raphe	DR
Ectorhinal area	ECT
Entorhinal area, lateral part	ENTl
Periaqueductal gray	PAG
Parabrachial nucleus	PB
Parafascicular nucleus	PF
Posterior triangular thalamic nucleus	POT
Pedunculopontine nucleus	PPN
Parasubthalamic nucleus	PSTN
Substantia nigra	SN
Subthalamic nucleus	STN
Temporal association areas	TEa
Postpiriform transition area	TR
Ventral posterior complex of the thalamus	VP
Ventral tegmental area	VTA

Supplementary Table 4. Additional information of riboprobes used for FISH.

Probe	Accession No.	Probe Region	Primer Sequence
CaMKII	NM_009792.3	1346–2219	Forward Primer: AGTCTCCAAGCCAACCCC<br>Reverse Primer: ATAGAGCGCACACCAGGC
GAD1	NM_001312900.1	1032–1522	Forward Primer: TCAGGGGACCTCCAAGGAAA<br>Reverse Primer: ATGGAAGCAAGATCGGGGTG
CRF	NM_205769.3	224–827	Forward Primer: GGAGAAGAGAGCGCCCCTA<br>Reverse Primer: TTTGGCCAAGCGCAACATTT

Supplementary Table 5. Parameters of measurements and descriptions used in L-Measure.

Measurement	Description
Soma surface	The surface of the soma
N stems	The number of stems attached to the soma
N bifs	The number of bifurcations of a given neuron
N branches	The number of branches in a given neuron
N tips	The number of terminal tips for a given neuron
Width	The span of the X-axis of a given neuron in space
Depth	The span of the Z-axis of a given neuron in space
Depth width ratio	The value obtained by dividing the depth by the width
Euclidean distance	The Euclidean distance of the farthest terminal tip with respect to the soma
Path distance	The sum of lengths of each compartment from the soma point to the farthest terminal tip
Branch path length	The sum of lengths of all compartments forming a given neuron

Supplementary Table 6. Primary antibodies and secondary antibodies used in the immunostaining procedure.

Primary Antibodies

Target	Host	Dilution	Company	Catalogue	Lot
DsRed	Rabbit	1:1,000	CLONTECH	632496	1612022
NECAB1	Mouse	1:500	ATLAS ANTIBODIES	AMAb90801	MAB-02714
PSD-95	Goat	1:500	ABCAM	ab12093	GR212888-1
MBP	Rabbit	1:500	DAKO	A0623	A0090A
GABA	Rabbit	1:500	Sigma	A2052-2ML	018M4808V

Secondary Antibodies

Target	Host	Fluorophore	Dilution	Company	Code
Rabbit	Donkey	Alexa Fluor 594	1:200	Jackson	711-585-152
Mouse	Donkey	Alexa Fluor 488	1:200	Jackson	715-545-150
Goat	Donkey	Alexa Fluor 488	1:200	Jackson	705-545-147
Rabbit	Donkey	Alexa Fluor 488	1:200	Jackson	711-545-152
