# Supplementary material for: Single-cell reconstruction reveals input patterns and pathways into corticotropin-releasing factor neurons in the central amygdala in mice
Source: Commun Biol. 2022 Apr 6;5:322. doi: 10.1038/s42003-022-03260-9 (PMC8986827; doi:10.1038/s42003-022-03260-9)
Supplement: Supplementary file 3 — Description of Additional Supplementary Files [file 42003_2022_3260_MOESM3_ESM.pdf]

Description of Additional Supplementary Files

Supplementary Movie 1: Three-dimensional reconstruction of input neurons at whole brain scale by VISoR light-sheet.

Supplementary Movie 2: The input neuron in somatosensory cortex and the en passant brain structures through which it projected to CeA-CRF neurons.

Supplementary Movie 3: The input neuron in mediodorsal nucleus of thalamus and the en passant brain structures through which it projected to CeA-CRF neurons.

Supplementary Movie 4: The input neuron in periaqueductal gray and the en passant brain structures through which it projected to CeA-CRF neurons.

Supplementary Data 1: The cell counts in all input brain subregions of all samples.
