# Supplementary material for: Single-cell reconstruction reveals input patterns and pathways into corticotropin-releasing factor neurons in the central amygdala in mice
Source: Commun Biol. 2022 Apr 6;5:322. doi: 10.1038/s42003-022-03260-9 (PMC8986827; doi:10.1038/s42003-022-03260-9)
Supplement: Supplementary file 9 — Reporting Summary [file 42003_2022_3260_MOESM9_ESM.pdf]

nature portfolio

Corresponding author(s): Jiang-Ning Zhou

Last updated by author(s): Feb 18, 2022

Reporting Summary

Nature Portfolio wishes to improve the reproducibility of the work that we publish. This form provides structure for consistency and transparency in reporting. For further information on Nature Portfolio policies, see our Editorial Policies and the Editorial Policy Checklist.

Statistics

For all statistical analyses, confirm that the following items are present in the figure legend, table legend, main text, or Methods section.

n/a	Confirmed	
☐	☒	The exact sample size (n) for each experimental group/condition, given as a discrete number and unit of measurement
☐	☒	A statement on whether measurements were taken from distinct samples or whether the same sample was measured repeatedly
☐	☒	The statistical test(s) used AND whether they are one- or two-sided<br>*Only common tests should be described solely by name; describe more complex techniques in the Methods section.*
☐	☒	A description of all covariates tested
☐	☒	A description of any assumptions or corrections, such as tests of normality and adjustment for multiple comparisons
☐	☒	A full description of the statistical parameters including central tendency (e.g. means) or other basic estimates (e.g. regression coefficient) AND variation (e.g. standard deviation) or associated estimates of uncertainty (e.g. confidence intervals)
☐	☒	For null hypothesis testing, the test statistic (e.g. F, t, r) with confidence intervals, effect sizes, degrees of freedom and P value noted<br>*Give P values as exact values whenever suitable.*
☒	☐	For Bayesian analysis, information on the choice of priors and Markov chain Monte Carlo settings
☒	☐	For hierarchical and complex designs, identification of the appropriate level for tests and full reporting of outcomes
☐	☒	Estimates of effect sizes (e.g. Cohen's d, Pearson's r), indicating how they were calculated

Our web collection on statistics for biologists contains articles on many of the points above.

Software and code

Policy information about availability of computer code

Data collection	TissueFaxs Plus system (Tissue Genostics)<br>LSM 880 (Zeiss)<br>VISoR (NEL-BITA)
Data analysis	Matlab 9.7 (Mathworks)<br>SPSS Statistics 22.0 (IBM)<br>Prism 8 (GraphPad)<br>Amira 6 (Thermo Fisher Scientific)<br>Imaris 9.3.1 (Bitplane)<br>3DS Max (Autodesk)<br>L-Measure<br>Lychin

For manuscripts utilizing custom algorithms or software that are central to the research but not yet described in published literature, software must be made available to editors and reviewers. We strongly encourage code deposition in a community repository (e.g. GitHub). See the Nature Portfolio guidelines for submitting code & software for further information.

Data

Policy information about availability of data

All manuscripts must include a data availability statement. This statement should provide the following information, where applicable:

- Accession codes, unique identifiers, or web links for publicly available datasets
- A description of any restrictions on data availability
- For clinical datasets or third party data, please ensure that the statement adheres to our policy

The data that support the findings of this study are available from the corresponding author upon request.

Field-specific reporting

Please select the one below that is the best fit for your research. If you are not sure, read the appropriate sections before making your selection.

☒ Life sciences ☐ Behavioural & social sciences ☐ Ecological, evolutionary & environmental sciences

For a reference copy of the document with all sections, see nature.com/documents/nr-reporting-summary-flat.pdf

Life sciences study design

All studies must disclose on these points even when the disclosure is negative.

Sample size	No statistical methods were used to predetermine sample size. The sample sizes were chosen based on published studies in the field. For references, see Statistical analysis paragraph in Methods section.
Data exclusions	The exclusion criteria were established prior to data collection:<br>Mice were post-hoc excluded from the analysis if more than 30% of the starters were outside of the central amygdala (CeA).<br>Data exclusion criteria are also described in detail in the respective Methods sections.
Replication	Results were reproduced by multiple rounds of experiments, i.e., from multiple mice. No results are included that were not observed in multiple animals.
Randomization	Animals were not randomized due to the necessity of a genetic construct (CRF-Cre mice).
Blinding	Investigators were not blind to subject groups because knowledge of experimental conditions was required during data collection and evaluation.

Reporting for specific materials, systems and methods

We require information from authors about some types of materials, experimental systems and methods used in many studies. Here, indicate whether each material, system or method listed is relevant to your study. If you are not sure if a list item applies to your research, read the appropriate section before selecting a response.

Materials & experimental systems

n/a	Involved in the study	
☐	☒	Antibodies
☒	☐	Eukaryotic cell lines
☒	☐	Palaeontology and archaeology
☐	☒	Animals and other organisms
☒	☐	Human research participants
☒	☐	Clinical data
☒	☐	Dual use research of concern

Methods

n/a	Involved in the study	
☒	☐	ChIP-seq
☒	☐	Flow cytometry
☒	☐	MRI-based neuroimaging

Antibodies

Antibodies used	Anti-DsRed primary antibody, CLONTECH, catalog number:632496<br>Anti-NECAB1 primary antibody, ATLAS ANTIBODIES, catalog number:AMAb90801<br>Anti-PSD-95 primary antibody, ABCAM, catalog number:ab12093<br>Anti-MBP primary antibody, DAKO, catalog number:A0623<br>Anti-GABA primary antibody, Sigma, catalog number:A2052-2ML<br>Donkey anti-rabbit secondary antibody AlexaFluor-594-conjugated, Jackson immoresearch, catalog number: 711-585-152<br>Donkey anti-mouse secondary antibody AlexaFluor-488-conjugated, Jackson immoresearch, catalog number: 715-545-150

	Donkey anti-goat secondary antibody AlexaFluor-488-conjugated, Jackson immoresearch, catalog number: 705-545-147<br>Donkey anti-rabbit secondary antibody AlexaFluor-488-conjugated, Jackson immoresearch, catalog number: 711-545-152
Validation	All primary antibody is a well characterized commercial antibody. The specificity of the primary and secondary antibodies was validated by the manufacturers.<br>reference:<br>Mauriac S, Hien Y, Bird J, Carvalho S, Peyroutou R, Lee S, et al. Defective Gpsm2/Gαi3 signalling disrupts stereocilia development and growth cone actin dynamics in Chudley-McCullough syndrome. Nat Commun. 2017;8:14907.<br>Burger CA, Alevy J, Casasent AK, Jiang D, Albrecht NE, Liang JH, Hirano AA, Brecha NC, Samuel MA. LKB1 coordinates neurite remodeling to drive synapse layer emergence in the outer retina. Elife. 2020 May 7;9:e56931. doi: 10.7554/eLife.56931.<br>Abdo H, Calvo-Enrique L, Lopez JM, Song J, Zhang MD, Usoskin D, El Manira A, Adameyko I, Hjerling-Leffler J, Ernfors P. Specialized cutaneous Schwann cells initiate pain sensation. Science. 2019 Aug 16;365(6454):695-699. doi: 10.1126/science.aax6452.

Animals and other organisms

Policy information about studies involving animals; ARRIVE guidelines recommended for reporting animal research

Laboratory animals	Mus musculus, males CRF-ires-Cre (background: C57BL/6, Jackson Laboratory, 012704), aged 2-3 months at the time of first injection (3-4 months at time of the experiment).
Wild animals	This study did not involve wild animals.
Field-collected samples	This study did not involve samples collected from the field.
Ethics oversight	All animal procedures were approved and conducted in accordance with the Institutional Animal Care and Use Committee at the University of Science and Technology of China.

Note that full information on the approval of the study protocol must also be provided in the manuscript.
